# Development and Validation of a New LC-MS/MS Method for the Assay of Plasmatic Peripheral Short- and Medium-Chain Fatty Acids for Metabolomics Applications

**DOI:** 10.3390/metabo15060403

**Published:** 2025-06-16

**Authors:** Lenard Farczadi, Laura Barcutean, Smaranda Maier, Rodica Balasa, Silvia Imre

**Affiliations:** 1 Chromatography and Mass Spectrometry Laboratory, Center for Advanced Medical and Pharmaceutical Research, George Emil Palade University of Medicine, Pharmacy, Science and Technology of Targu Mures, 540139 Targu Mures, Romania; lenard.farczadi@umfst.ro (L.F.);; 2 Department of Neurology, Faculty of Medicine, George Emil Palade University of Medicine, Pharmacy, Science and Technology of Targu Mures, 540142 Targu Mures, Romania; 3 Neurology 1 Clinic, Mures County Emergency Clinical Hospital, 540136 Targu Mures, Romania; 4 Department of Analytical Chemistry and Drug Analysis, Faculty of Pharmacy, George Emil Palade University of Medicine, Pharmacy, Science, and Technology of Targu Mures, 540142 Targu Mures, Romania

**Keywords:** multiple sclerosis, short-chain fatty acids, LC-MS/MS, plasma

## Abstract

*Background:* Short-chain fatty acids (SCFAs) and medium-chain fatty acids (MCFAs) are human metabolites which are involved in various biochemical processes and can offer valuable insights and information on various pathological and metabolic issues of patients. Accurate, precise, high-performance bioanalytical methods are important tools in both research and diagnostics of many pathologies, with LC-MS being the most frequently used methodology in modern metabolomics studies. *Methods:* The current paper describes a complete LC-MS/MS methodology for the accurate quantification of total plasmatic SCFA concentrations in humans using high-resolution QTOF mass spectrometric detection, including sample cleanup, preparation, and derivatization. *Results and Conclusions:* The method was validated with regard to all relevant parameters (selectivity, sensitivity, accuracy, precision, linearity, recovery, carryover, and reproducibility of sample preparation) according to the current applicable guidelines and tested in an in vivo study to quantify peripheral SCFAs in human patients as biomarkers for gut–brain axis disruption.

## 1. Introduction

The use of biological molecules, products, or precursors in metabolic processes plays an essential role in clinical laboratory diagnosis or in measuring the status of pathological evolution or medical treatment. These molecules represent a wide range of chemical structures and they are found in various biological environments, which are challenging due to matrix complexity from the perspective of analytic methodology. As small molecules with a hydrophilic nature, these endogenous compounds represent a challenge for analytical determination and quantification, when compared to, for example, non-endogenous compounds or compounds which are more hydrophobic in chemical nature, and thus are more separated through chromatographic methods and are less likely to necessitate chemical derivatization. Moreover, in certain situations, such as analyzing samples with very low levels of concentrations of analytes of interest, clinical laboratory determinations are increasingly turning towards analytical methods with detectors, which meet the higher criteria of specificity/selectivity/sensitivity while also allowing for high-throughput analysis, essential in a clinical setting.

Immunological methods, such as ELISA, which are often found in clinical laboratories, while having some limitations, remain extremely cost-efficient, accessible, and useful, which explains their widespread use. This wide acceptance and availability of such methods in clinical analysis was achieved through equipment standardization and automation of working methods, resulting in a decrease in costs per analysis in comparison with other methods, such as chromatographic methods coupled with mass spectrometry. Although the latter have clearly superior specificity/selectivity and even sensitivity, they are not yet mandatory for most determinations in a clinical lab facility.

Short-chain fatty acids are biological markers with relevance in clinical practice covering various pathologies, including hypertension, renal and hepatic diseases, multiple sclerosis, etc., and these acids playing an important role in the modulation of physiological processes.

SCFAs are molecules produced by the gut microbiome, mainly by metabolization of dietary fibers. Among them, there are three which are predominantly formed, acetic (AA), propionic (PA), and butyric (BA) acids. Their positive biological activity is closely linked to the fine equilibrium between their concentrations in the intestine [1].

The biological effects of SCFAs include anti-inflammatory, immunoregulatory, anti-tumoral, anti-diabetic, cardiovascular, hepatic, and neurological protective effects. According to the literature, lowering blood lipid levels and antibacterial activity are other benefits of these acids [2,3,4]. In general, these molecules are intensively studied from multiple other perspectives, one of these being associated with the understanding of the bidirectional communication between the microbiome and human cells, such as quorum sensing (QS) systems with a role in the regulation and signaling functions of the microbiome [5].

Nevertheless, there are studies in which side effects of some SCFAs are also investigated, but these are shown to depend on the type of acid, concentration, or pathological complexity of the human study subjects [1].

The determination of SCFAs in biological matrices by chromatographic methods coupled with mass spectrometry is the standard approach in the research of these molecules due to the advantage in analytical performance this technique offers. The methods described in the literature are generally based on chemical pre-treatment for derivatization of the acids in order to improve their chromatographic retention and mass spectrometric detection. An important number of LC-MS methods has been published in which nitrogen-containing compounds—amines, hydroxylamines, nitrophenylhydrazines, benzoxadiazoles, cyclic amines, etc.—are used for derivatization of SCFAs [6,7,8,9,10].

On the other hand, there are studies in which analysis of SCFAs without chemical derivatization is proposed, such as the study of Lee et al., published in 2024. In this case, a poly(vinyl alcohol)-based chromatographic column is used, and the mechanism of separation is based on the principle of steric exclusion [11]. In another study, after sample purification by liquid–liquid extraction, LC-MS analysis was performed in the reversed mode and without chemical derivatization of the analytes. This method allows for the quantification of multiple types of molecules, SCFAs, bile acids, and ketoacids [12].

The great interest in developing analytical methods for fatty acid determination is also underlined by the research of Jiao et al., in which a computational study was carried out, analyzing short-, medium-, and long-chain fatty acids via LC-MS determination of the acids after derivatization with 3-nitrophenylhydrazine [13].

Despite the rather large number of LC-MS methods published in the literature, not all methods can be reproduced with fidelity, either due to differences between the types of detectors used, the complexity of the sample matrices to be analyzed, the profile of acids to be determined, or, last but not least, the accessibility of derivatization reagents. On the other hand, the functional and performance differences between low-resolution mass spectrometric detectors (ion trap, triple quadrupole) and high-resolution MS (QTOF, Q-Orbitrap) can also become a hurdle standing in the way of LC-MS method development, and they justify the availability of a great variety of methods published in literature. While high-resolution MS offers useful structural information on analytes, and it appears almost a mandatory technique to identify analytes of interest in a complex biological matrix, it is not as widely available as low-resolution MS. Besides the wider availability, the substantially lower costs of acquisition and use of low-resolution mass spectrometers make them a viable option for analysis where the higher selectivity and sensitivity of high-resolution MS are not mandatory, and as such explain the higher number of publications using low-resolution MS compared to the ones using high-resolution MS [14].

Taking into consideration the above aspects, the aim of the present study was to develop and validate an in-house LC-QTOF mass spectrometry method for the determination of acetic, propionic, butyric, and caproic acids in plasma after chemical derivatization. The methodology was developed in order to allow the estimation of levels and concentration ratios between the considered acids in an accurate and precise manner after simple sample purification handling by protein precipitation, which was proven to have the necessary selectivity and sensitivity for the determination.

## 2. Materials and Methods

### 2.1. Reagents

HPLC-grade acetonitrile was purchased from Riedel-de Haen, and HPLC methanol was purchased from VWR, while analytical-grade formic acid was purchased from Merck. Ultrapure water was produced in-house using a Millipore DQ-3 system. Pyridine, 3-nitrophenylhydrazine, and 1-ethyl-3-(3-dimethylaminopropyl)carbodiimide for SCFA derivatization were all purchased from Sigma-Aldrich. Analytical standards for acetic acid (100%, Cat No. 71251-5ML-F), propionic (propanoic) acid (100%, Cat No. 94425-5ML-F), butyric (butanoic) acid (100%, Cat No. 19215-5ML), and hexanoic (caproic) acid (100%, Cat No. 21529-5ML) were all acquired from Sigma Aldrich (Burlington, VT, USA). The internal standard, deuterated hexanonic-d3 (caproic-d3) acid, was purchased from Sigma Aldrich (≥99%, Cat No. 489727-100 mg). Plasma samples and blanks were obtained from clinical studies (published separately), all of which were subject to ethics committee reviews and received approvals.

### 2.2. Equipment

LC-MS/MS analysis was performed on a system consisting of an AB Sciex (Framingham, MA, USA) 4600 QTOF type mass spectrometer coupled with a Flexar FX-10 UHPLC liquid chromatograph from Perkin Elmer (Shelton, CT, USA). We utilized vortex mixers made by Velp Scientifica (Usmate Velate, Italy) and Heidolph (Schwabach, Germany), along with centrifuges manufactured by Eppendorf (Hamburg, Germany). Other equipment used was as follows: Radwag analytical balance (Radom, Poland), JP Selecta ultrasonic bath (Barcelona, Spain), Eppendorf automatic variable volume single-channel pipettes (Hamburg, Germany), and Millipore DQ-3 water ultra-purifying system (Burlington, VT, USA).

### 2.3. LC-MS Analysis Method

Chromatographic separation of the derivatized short-chain fatty acids was performed using a Gemini NX-C18 column with the dimensions 3.0 × 100 mm (particle diameter 3 μm) and a mobile phase composed of 0.2% (*v*/*v*) formic acid and acetonitrile in a ratio of 70:30 (*v*/*v*) in isocratic elution, with a constant flow rate of 0.5 mL/min. The analytical column was thermostatted at 25 °C, while the samples were kept in the autosampler at 20 °C. The injection volume was 10 μL per sample for analysis of plasma samples (standard solutions, quality control solutions, and biological samples), and all analytes were chromatographically separated after a total run time of 18 min per sample.

The derivatized SCFAs and internal standard were separated chromatographically ad then identified and quantified by negative electrospray ionization (ESI-), followed by MS/MS MRM mass spectrometric detection by specific fragmentation monitoring for each analyte and the internal standard. The ionization source parameters for optimal ionization were a spray voltage = −4500 V, vaporizer temperature = 500 °C, ion gas source 1 = 20 bar, ion gas source 2 = 25 bar, and curtain gas = 30 bar. For the identification and quantification of analytes, specific fragment patterns and *m*/*z* (mass-to-charge ratios) were used for each analyte, with multiple fragments summed for each individual analyte in order to increase peak intensity and thus the sensitivity of the method. The fragments used for quantification, arranged by intensity, along with retention times of the analytes are described in Table 1.

### 2.4. Calibration and Quality Control (QC) Standard Solutions for Plasma Sample Analysis

For the quantification of the analytes, the internal standard method was applied using plasma calibration curves prepared using certified reference substances. Stock solutions were prepared in a mixture of water:methanol 3:7 (*v*/*v*) with concentrations of 1 mg/mL and 10 µg/mL for each SCFA, as well as a stock solutions of internal standard in methanol with 20 mg/mL and 10 µg/mL caproic-d3 acid. Stock solutions were used and further diluted with a mixture of water:methanol 3:7 (*v*/*v*) to prepare a working solution mixture containing all SCFAs with a concentration of 1000 ng/mL for each analyte, after which five further working solutions containing mixtures of all analytes were prepared using the same dilution solvent (water:methanol 3:7 (*v*/*v*)), with concentrations between 10 and 500 ng/mL. The working solutions were further used for the calibration curve standard solutions. The calibration standard solutions with a nominal concentration range of 10–1000 ng/mL were prepared by mixing 100 µL of each working solution with 10 µL of internal standard solution (10 µg/mL caproic-d3 acid), 50 µL of EDC solution (50 mM in water:methanol 3:7 (*v*/*v*)), 50 µL of 3-NPH solution (50 mM in water:methanol 3:7 (*v*/*v*)), and 50 µL of PYR solution (7% (*v*/*v*) in water:methanol 3:7 (*v*/*v*)). The mixture was mixed for 1 h at 800 rpm, after which 250 µL of aqueous 0.5% formic acid solution (*v*/*v*) was added and the samples were transferred to chromatographic vials. The lower limit of quantification was set at 10 ng/mL for each analyte. Separately, working solutions for quality control samples were prepared at the same six concentration levels (QCA, QCB, QCC, QCD, QCE, QCF). These were processed identically to calibration curve standards and used during the validation of the method and for quality control of biological sample analysis sequences. Calibration curves were constructed automatically by Analyst 5.0 software with a linear fit and 1/y^2^ weighing.

### 2.5. Preparation of Biological Plasma Samples

For the quantitation of free plasma SCFAs, 50 µL of plasma sample was precipitated with 150 µL of methanol, vortexed for 1 min at 2000 rpm, and then centrifuged at 9900 g rcf for 5 min. We then mixed 100 µL of supernatant with 10 µL of internal standard solution (10 µg/mL caproic-d3 acid), 50 µL of EDC solution (50 mM in water:methanol 3:7 (*v*/*v*)), 50 µL of 3-NPH solution (50 mM in water:methanol 3:7 (*v*/*v*)), and 50 µL of PYR solution (7% (*v*/*v*) in water:methanol 3:7 (*v*/*v*)). The mixture was vortexed for 2 min at 2000 rpm and then centrifuged at 9900 g rcf for 10 min. The mixture was next mixed for 1 h at 800 rpm, after which 250 µL of aqueous 0.5% formic acid solution (*v*/*v*) was added, and the samples were transferred to chromatographic vials and injected into the LC-MS/MS system.

### 2.6. Validation of the Analytical Method

The bioanalytical method for SCFA quantification from plasma was validated by taking into consideration all relevant performance parameters in order to ensure the results obtained were reliable. The method was validated according to current international validation guidelines [15,16] with regard to all parameters relevant for the use of the method for this application.

The method was validated with regards to selectivity and sensitivity by comparing the peak area at the lower limit of quantification for each derivatized analyte with possible interfering peaks appearing in blank solvent (water:methanol, 3:7 (*v*/*v*)) solutions’ chromatograms after derivatization and sample preparation procedures, on mass-specific extracted chromatograms at the same retention times as analytes. Carryover of the method was tested by re-injecting the same blank solvent solutions after the highest-concentration calibration standard solution and checking for interfering peaks at the retention times of derivatization.

The linearity of the method was verified by plotting calibration curves for each derivatized analyte and using a linear fit with 1/y^2^ weighing. Calibration curves were then checked for their correlation coefficient (R) and accuracy of recalculated concentrations for the standard solutions.

The accuracy and precision of the method were validated by separately (from the calibration curve) injecting multiple quality control solutions (five of each of the concentration standards), both within the same analytical run for within-run validation as well as during multiple analytical runs for between-run accuracy and precision. The accuracy of each quality control standard was calculated as the bias (%) of the calculated concentration compared to the theoretical concentration, while precision was calculated as the coefficient of variation (CV, %) in the calculated concentration for all the QC standards at each concentration level.

Recovery of derivatized analytes from plasma was determined by comparing derivatized blank plasma samples with the sample blank plasma spiked with calibration standard solutions at two different concentration levels (50 ng/ml and 500 ng/ml for each analyte), which after spiking were also derivatized. As such, two types of solutions were prepared in order to determine the total analyte recovery: blank plasma samples (mixture of 150 µL of methanol and 50 µL of plasma, for which 100 µL of supernatant is mixed with 10 µL of internal standard solution and derivatized with EDS, 3-NHP, and PYR as described in chapter 2.5); 50 µL–50 µL (1:1, *v*/*v*) mixture of the same blank plasma (supernatant after the precipitation step with methanol) with 50 µL of standard mixture containing all SCFAs, which is then further mixed with 10 µL of internal standard solution and derivatized with EDS, 3-NHP, and PYR, as described in Chapter 2.5. The recovery was calculated as the bias (%) of the concentration of the spiked (theoretical concentration after spiking) and unspiked plasma for each analyte.

Additionally, the method was validated with regards to the reproducibility of the derivatization of SCFAs by derivatizing 4 parallel samples from the same pool of blank plasma, using two different sources of plasma, and calculating the coefficient of variation (CV, %) in the measured peak areas. This was repeated on a second day to ensure the within-run reproducibility of the derivatization is stable, as well as performing individual blank plasma samples from the same 2 pools of plasma on another 3 different days (one daily) in order to determine the within-run reproducibility for the 5-day workweek.

## 3. Results

**Figure 1:**
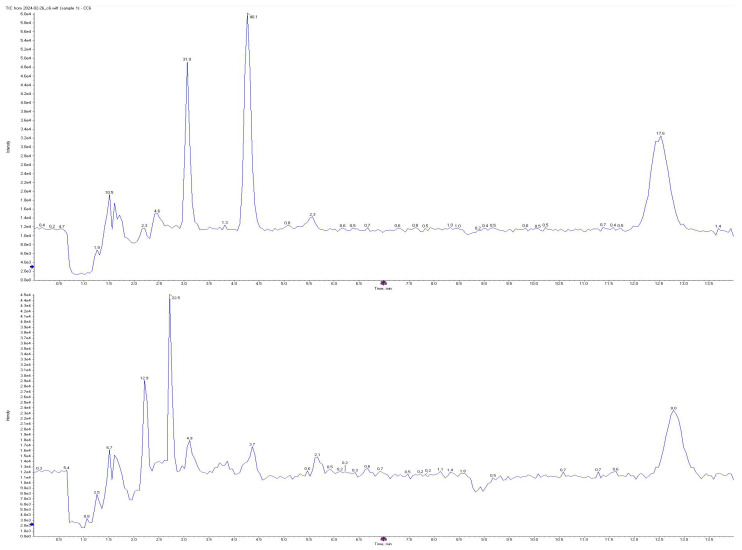
Overlayed total ion chromatograms (TIC) of AA, PA, BA, and CA standard solution (**above**) and plasma sample (**below**).

**Figure 2:**
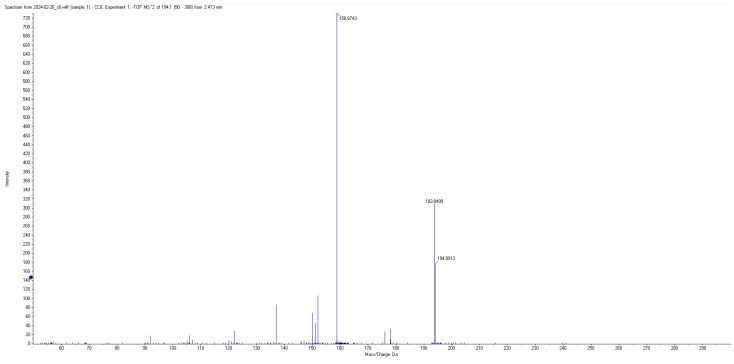
Mass spectra of derivatized AA.

**Figure 3:**
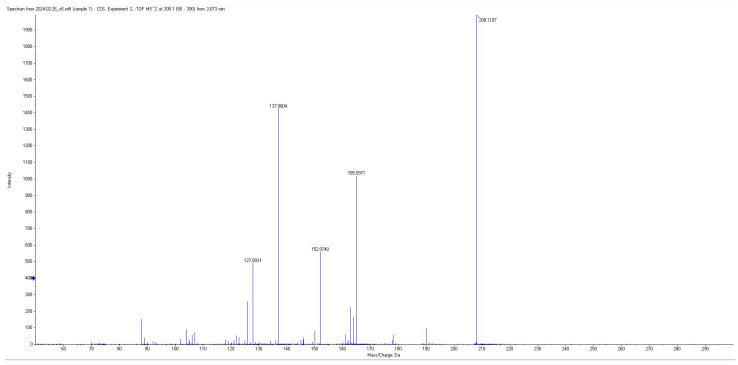
Mass spectra of derivatized PA.

**Figure 4:**
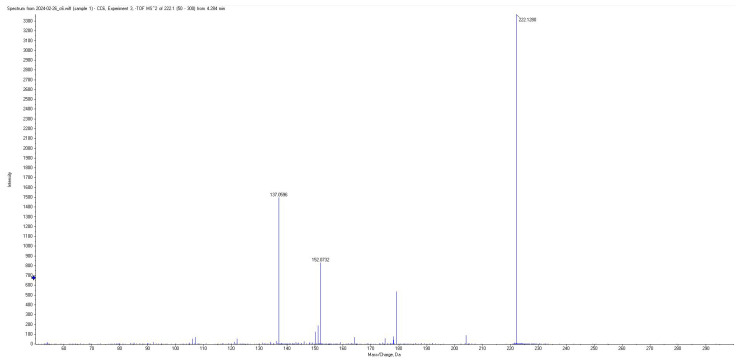
Mass spectra of derivatized BA.

**Figure 5:**
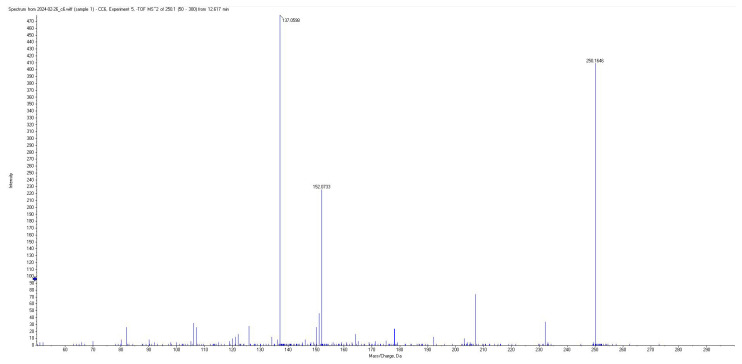
Mass spectra of derivatized CA.

All short-chain fatty acids were separated chromatographically (Figure 1) as well as based on their specific *m*/*z* mass fragmentation patterns (Figure 2, Figure 3, Figure 4 and Figure 5). The method was validated according to the methodology described, and all validation parameters were within acceptance criteria described by both European and American validation guidelines [15,16].

Fragmentation patterns and parameters were optimized in such a way as to obtain a limited number of fragments with high intensity, for maximum sensitivity and selectivity of the method, and reduced intensity for the parent pseudomolecular ion (Figure 2, Figure 3, Figure 4 and Figure 5) for each individual analyte, as well as for the internal standard.

### 3.1. Sensitivity and Selectivity

No interfering peaks with peak areas larger than 20% of the peak area at the lower limit of quantification (LLOQ) were detected for any of the derivatized FAs in the blank solvent samples. As such, the method can be considered sensitive and selective enough with regards to the blank samples tested and for the purpose it was developed for.

### 3.2. Carryover

Similarly, as with the sensitivity testing, after reinjecting the blank solvent samples after the calibration standard with the largest concentration (at the upper limit of quantification—ULOQ), there were no peaks detected at the retention times of the analytes. As such, there is no detectable carryover effect when using the method.

### 3.3. Linearity

For all calibration curves injected during the method validation, the mean curve was plotted using a linear fit and 1/y^2^ weighing. The coefficient of correlation (R) was larger than 0.99 for all calibration curves, and thus all calibration curves for each analyte are considered linear (Table 2).

For each of the analytes, the calibration curves had no calibration standards that needed to be excluded from the final curve due to accuracy issues. All calibration standards used during the entire validation process had a recalculated concentration with accuracy within the ±15% limit described in the guidelines (Table 3).

### 3.4. Accuracy and Precision

The accuracy and precision of the method were determined at three different concentration levels, which cover the calibration range. All mean accuracy values and precision values for quality control standard solutions were within the ±15% acceptance criteria described in the guidelines for all analytes and for both within-run, as well as between-run, accuracy and precision. Thus, the method can be considered accurate and precise for the applications it has been developed for (Table 4 and Table 5).

### 3.5. Recovery for Plasma Sample Analysis

After analyzing blank plasma solutions and the same plasma spiked with standard solutions, the recovery was calculated as the bias (%) of the concentration of the spiked and unspiked plasma solutions. The results proved that the recovery of all the analytes was within ±15% after plasma protein precipitation and derivatization, and thus the derivatization yield results make the method suitable for this application. The results of the recovery testing are shown in Table 6.

### 3.6. Reproducibility of Plasma Sample Preparation for Total PUFA Analysis

The validation of the reproducibility of the sample derivatization and preparation method for SCFA/MCFA determination was performed by determining the coefficient of variation (CV, %) for blank plasma samples derivatized and prepared as described in Section 2.5 for both within-run and between-run sample preparation. The results showed that the method yields consistent and reliable results, and there is no significant variation during sample derivatization and preparation between the different plasma samples, regardless of whether prepared within one batch or individually (daily) over a period of multiple days (Table 7).

## 4. Discussion

There are a number of published analytical methods that demonstrate the potential of LC-MS/MS as a reliable and robust technique for the determination of various fatty acids, including SCFAs and MCFAs, from various relevant biological samples. The existing studies describe different strategies regarding derivatization, chromatographic separation, and sample preparation techniques. Regarding the type of mass spectrometer used for determinations, there have been methods described mainly using triple quadrupole but also ion trap or orbitrap mass spectrometers. There have even been methods described using gas chromatography coupled with mass spectrometry.

While there are already a number of analytical methodologies described in the scientific literature, including sample cleanup, preparation, and derivatization techniques, as well LC-MS methods, it is important to note that the existing methodologies cannot always be directly implemented and can be reproduced with fidelity, due to differences in mass spectrometers and mass detectors, liquid chromatographs, or even auxiliary laboratory equipment available, alongside other factors. Nagatomo et al. describe and compare some of the different sample preparation methods described in the literature, and while they conclude that they generally all work for their intended purpose, they discuss the differences in performance and the advantages/disadvantages each method provides [17]. Similarly, the different types of mass spectrometric and chromatographic methods might offer advantages and disadvantages, and certain methods might suit some laboratories and users better than, others depending on equipment availability, funding, experience know-how of the analyst, etc. Chalova et al., in their systematic review of methods for SCFA/MCFA analysis, came to the same conclusion, that the wide abundance of methods is justified by the ever-increasing importance of metabolomics studies in cancer research as well as other types of medical research, pointing out that the variety of analytical methodologies (gas chromatography, liquid chromatography, and even capillary electrophoresis) each come with advantages and disadvantages but cover a wide range of practical analytical needs [18]. At the same time, Chalova et al. point out the importance of choosing the most suitable sample preparation (including derivatization, if necessary) method on a case-by-case basis to improve efficiency and applicability depending on the specifics of each study [18].

By using the derivatization method described by Cheng Li et al. [19] as a starting point, which was originally developed for biological samples of animal origin, we managed to optimize the sample preparation and cleanup for human plasma samples and at the same time were able to adapt the technique to better suit the available laboratory equipment, while at the same time trying to improve applicability, efficiency, and reproducibility. Compared to the method described by Li et al., we added a deuterated internal standard to not only improve the reproducibility but also better comply with international regulatory guidelines, and we reduced the analysis time and reduced the column temperature to improve column stability.

Chen et al. describe a new LC-MS method for the quantification of two fatty acids, butanoic acid and caproic acid, from serum, using liquid–liquid extraction and derivatization with 2-nitrophenylhydrazine, undecanoic acid as an internal standard, and a 10 min gradient [20]. This method, while fit for its purpose, has the disadvantage of quantifying only two analytes, with a comparatively long analysis time compared to the method described in the current manuscript, which quantifies four analytes using a gradient which only needs three additional minutes of analysis time to completely separate and elute all analytes. At the same time, the liquid–liquid extraction step, compared to simple protein precipitation, needs additional time to prepare each sample and uses hazardous n-hexane as an extraction solvent. Additionally, the method described by our research team uses a deuterated internal standard, which while costly, assures higher reproducibility, easier LC-MS method development, better compliance with regulatory guidance, and more reliable results.

Dei Cas et al. described a very similar LC-MS method, also using derivatization with 3-NPH after protein precipitation treatment of samples (which is performed with 2-propanol instead of methanol) [21]. However, due to financial restrictions for this method, Dei Cas et al. were not able to use an isotopically labelled internal standard, mentioning that this would have been the ideal solution, but instead used an iso-butoxy analog of acetic acid, which they found the most acceptable alternative from a wide range of compounds tested [21]. The analysis runtime for each sample is also longer, with the chromatographic gradient for each sample taking 18 min.

In another study very similar to our own, a new LC-MS method for the quantification of the same range of fatty acids, as well as a few of their metabolites, was developed by Jaochico et al. [22] using a different derivatization agent, O-benzylhydroxylamine. While this methodology was optimized for very efficient chromatographic analyte separation, with all analytes being separated using a gradient taking less than 8 min per sample, and uses isotopically labeled internal standards for excellent reproducibility and reliability, it does have the disadvantage of using a more complex sample cleanup with liquid–liquid extraction and has reduced sensitivity (LLOQ of 25.2 ng/mL for each analyte) compared to the methodology developed by our team [22]. Building on this method developed by Jaochico et al. [22], Vagaggini et al. further developed and optimized the technique for different types of plasma (rat plasma), further proving the versatility and utility of LC-MS methods, as well as how a given method can be optimized and adapted for use in animal research studies after being developed for human samples, and vice versa [23].

Mayo-Martinez et al. also had a slightly different approach for analyzing the same range of SCFAs and MCFAs, using another derivatization method previously described in the literature with Dansyl Hydrazine [24]. This method had the disadvantage of a significantly longer analysis time (around 20 min per sample) due to the modified retention of the derivatized fatty acids, as well as the fragmentation patterns, where all analytes and internal standards yielded the same single monitored fragment ion (*m*/*z*), which could cause selectivity issues with analysis of isomeric compounds, and as such greater care must be taken with chromatographic separation.

Saha et al. described another LC-MS method for the direct, underivatized quantification of SCFAs [25]. The technique described by Saha et al. settles on using a porous graphitic carbon analytical column, after testing a number of specialized polar columns. Although sample preparation is greatly simplified and sample preparation times reduced, due to the physical–chemical properties of the analytes, chromatographic separation is more difficult and necessitates more expensive, less stable chromatographic columns [25]. While this approach is definitely a good addition for the scientific community, it does not come without its downsides compared to the previously described methodologies using chemical derivatization of SCFA/MCFA.

Another method using derivatization with 3-NPH was described in a manuscript by Shafaei et al., which was also fully validated by the research team [26]. Highly sensitive and selective, with a short analysis time, this method uses a rather cumbersome sample preparation method with two-step liquid–liquid extraction using methyl tert-butyl ether.

In an attempt to improve the ionization of SCFAs and MCFAs during LC-MS analysis, Song et al. derivatized the analytes using Girard’s reagent T (trimethylaminoacetohydrazide chloride) [27]. This derivatization did offer excellent selectivity and sensitivity by improving fragmentation patterns and ionization; however, this approach resulted in a novel LC-MS technique, which still necessitates polar chromatographic columns as underivatized fatty acids, and in the end, leads to a chromatographic separation of derivatized fatty acids, which was not ideal, despite the rather long sample analysis time of almost 20 min.

In a novel approach, Li et al. synthetized sulfonyl piperazinyl analogs for a new type of SCFA and MCFA derivatization method, followed by LC-MS analysis [28]. While this approach is indeed novel and offers a new, additional type of derivatization for SCFA/MCFA analysis, it also comes with some drawbacks compared to the more established previous derivatization methods. One aspect to consider is the current unavailability of the derivatization agents used by Li et al., as they were completely synthetized by the research team, and as such, at the moment this reaction is not a widely applicable solution for FA analysis [28]. At the same time, while this derivatization is reported by the authors to greatly improve chromatographic retention, and as such is a good option for separating a larger number of analytes, it does have the downside of necessitating an analysis time of nearly 60 min per sample and a very high column temperature, thus reducing the practicality in high-throughput metabolomics studies compared to the more traditional LC-MS methods described previously in the literature, as well as in the current manuscript.

To further outline the importance of SCFA and MCFA metabolomics studies, the research team of Wang et al. developed a mathematical model and computational simulation for the analysis of a very large number of short-, medium-, long-, and very-long-chain fatty acids through LC-MS after 3-NPH derivatization. The model was validated with a restricted number of fatty acids for calculating the simulated retention times on a C18 chromatographic column, as well as the collision energies and fragmentation patterns [13]. This model could in conjunction with a well-established LC-MS method allow for the analysis of a wide range and large number of fatty acids from plasma samples, after derivatization with 3-NPH, without the need for analytical standards, thus reducing the cost and increasing the versatility for any methods using this type of sample preparation and derivatization, including the current research presented in this manuscript, highlighting the potential of the methodology developed.

The method described in this manuscript has been specifically developed with ease of use in mind, and as such, sample preparation and derivatization have been developed to be as easy to apply as possible. The relatively easy sample preparation protocol combined with the short run time per sample mean the method is suitable for a clinical setting, offering simple, high-throughput quantification. At the same time, the mass spectrometric detection was optimized for the best performance on high-resolution detection, while being adaptable to any mass spectrometer (including low-resolution mass detectors). The sample cleanup and derivatization process proved to be reproducible and yielded reliable results, while the complete analytical separation of analytes was performed while maintaining a high accuracy and precision of measurements, below the ±15% threshold described in bioanalytical validation guidelines [15,16]. Linearity, selectivity (complete lack of interfering peaks), and recovery testing proved that the method is suitable for the quantification of SCFAs and MCFAs from plasma obtained from patients, within biomonitoring, clinical studies, diagnostics, or other applications. As such, the method described, while not a novel method, still is a method which offers advantages over other similar methods published previously, possibly useful to scientists depending on the equipment and know-how available to them, and has the added advantage of having been proven fit for purpose—not only fully validated but also applied in clinical studies.

While a great variety of methods have been described in the literature for the quantification of SCFAs and MCFAs, each of them have their own advantages and disadvantages. The large number of published methods could indicate a lack of novelty; however, each research team optimized and adapted the techniques for their specific application and equipment, with each new method published bringing new optimization steps and small advantages compared to the existing knowledge. However, novel methods and approaches do not necessarily outperform previous, well-established techniques and methods. Our team took the same approach as most of the research groups, aiming to further optimize the methodologies and techniques while at the same time adapting a method for our specific clinical and research applications, and as such, the method described in this current manuscript brings a number of advantages. For instance, it was developed and optimized with simplicity in mind and was validated using all relevant parameters outlined by analytical validation guidelines [15,16], and it was adapted to ensure its usability in a real-life laboratory environment for all types of LC-MS users, regardless of previous experience, increasing usability in metabolomics studies. Sample preparation and derivatization were optimized in order to allow for quick and simple analysis, with a good reproducibility and accuracy, allowing for simple sample preparation with all widely available laboratory equipment. Sample derivatization was optimized in such a way as to yield solid and reproducible amounts of derivatized SCFAs, with reduced chances of errors or deviations. The larger number of research papers and methods published in the last few years show an increase in interest in the field of SCFA analysis and specifically the use of LC-MS for this purpose, and the new LC-MS/MS methodology developed by our team, including sample derivatization, fits into this trend, having been proven to be simple to use and having been successfully applied in a number of clinical metabolomics studies, some already published [6,29] and all carried out with the approval of an ethics committee, conducted in accordance with the principles outlined in the Declaration of Helsinki (1975, revised in 2013), and performed after obtaining written informed consent statements from all participants.

## Figures and Tables

**Table 1:** Mass spectrometric fragments monitored for analyte quantification.

SCFA	Molecular Weight (g/mol)	Parent Ion (*m*/*z*)	Fragment Ions (*m*/*z*)	Collision Energy (V)	Retention Time (min)
AA	60	194.07	152.05; 137.05; 122.02; 178.07; 150.05	−20	2.2
PA	74	208.09	152.05; 137.05; 150.05	−20	2.7
BA	88	222.07	152.05; 137.05; 122.02; 178.07; 150.05	−20	4.0
CA	116	250.13	152.05; 137.05; 178.07	−20	13.0
CA-d3 (internal standard)	119	253.14	155.05; 140.05; 181.07	−20	13.0

**Table 2:** Value of the coefficient of correlation for the calibration curves.

Batch	AA	PA	BA	CA
Batch 1	0.99808	0.99430	0.99769	0.99831
Batch 2	0.99476	0.99502	0.99490	0.99686
Batch 3	0.99816	0.99672	0.99853	0.99966
Batch 4	0.99916	0.99817	0.99860	0.99893
Batch 5	0.99865	0.99934	0.99785	0.99897

**Table 3:** Accuracy interval for calibration curve standard solutions.

	AA	PA	BA	CA
Accuracy (%)	−12.6 ÷ 14.5	−13.7 ÷ 14.0	−14.7 ÷ 11.3	−10.1 ÷ 9.3

**Table 4:** Within-run accuracy and precision for plasma QC analysis.

QC Solution	AA		PA		BA		CA	
	Accuracy (%)	Precision (%)	Accuracy (%)	Precision (%)	Accuracy (%)	Precision (%)	Accuracy (%)	Precision (%)
QCA	2.8	8.4	−6.8	11.3	8.4	3.9	3.8	7.5
QCB	6.5	8.6	−8.4	4.8	−7.5	5.0	1.2	8.1
QCC	−4.3	3.7	−5.3	5.2	−1.7	1.2	−4.8	3.3
QCD	−7.5	4.3	1.4	6.3	3.1	1.6	−0.6	2.2
QCE	8.2	5.6	8.1	2.5	5.5	2.0	3.0	2.2
QCF	3.0	1.9	12.6	2.1	5.0	2.4	6.3	1.8

**Table 5:** Between-run accuracy and precision for plasma QC analysis.

QC Solution	AA		PA		BA		CA	
	Accuracy (%)	Precision (%)	Accuracy (%)	Precision (%)	Accuracy (%)	Precision (%)	Accuracy (%)	Precision (%)
QCA	1.6	7.2	3.3	11.9	3.1	8.3	3.5	11.6
QCB	4.1	11.4	−6.2	6.3	−7.9	5.4	2.8	7.9
QCC	−2.3	1.1	−6.2	4.9	−1.1	3.3	−4.1	3.3
QCD	−5.7	5.8	0.4	5.3	2.2	1.8	−2.1	2.6
QCE	6.9	6.4	7.4	6.6	4.0	2.0	3.2	3.8
QCF	−0.9	3.1	6.4	1.4	4.4	2.4	4.0	2.9

**Table 6:** Recovery of SCFAs.

		Within-Run			Between-Run		
SCFA	Spike Std. Conc. (ng/mL)	Mean Conc. Plasma (ng/mL)	Mean Conc. Spiked Plasma (ng/mL)	Mean Recovery (%)	Mean Conc. Plasma (ng/mL)	Mean Conc. Spiked Plasma (ng/mL)	Mean Recovery (%)
AA	50	905.97	431.18	90.22	849.08	425.51	94.65
	500	905.97	763.76	108.64	849.08	742.11	110.02
PA	50	40.42	41.70	92.24	42.20	42.27	92.78
	500	40.42	308.38	114.13	42.20	301.44	111.19
BA	50	36.46	40.10	92.76	37.51	39.08	89.32
	500	36.46	302.87	112.91	37.51	298.31	110.99
CA	50	7.68	30.34	104.87	7.22	32.60	113.96
	500	7.68	286.72	112.95	7.22	282.70	111.47

**Table 7:** Reproducibility of the sample preparation and derivatization method.

	CV (%)		
SCFA	Within-Run (Day 1)	Within-Run (Day 2)	Between-Run
	[Plasma 1/Plasma 2]	[Plasma 1/Plasma 2]	[Plasma 1/Plasma 2]
AA	9.6/2.4	9.4/2.3	6.7/2.6
PA	7.8/14.5	6.5/12.7	5.2/11.9
BA	9.8/2.5	10.0/2.6	7.2/8.6
CA	11.2/13.4	10.5/10.8	14.1/13.9

## Data Availability

Data is unavailable due to privacy, intellectual property rights, or ethical restrictions.

## Abbreviations

The following abbreviations are used in this manuscript:

SCFA	Short-chain fatty acids
MCFA	Medium-chain fatty acids
MS	Multiple sclerosis
LC-MS	Liquid chromatography coupled with mass spectrometry
LC-MS/MS	Liquid chromatography coupled with tandem mass spectrometry
HPLC	High performance liquid chromatography
3-NPH	3-Nitrophenylhydrazine
EDC	1-Ethyl-3-(3-dimethylaminopropyl)carbodiimide
PYR	Pyridine
AA	Acetic acid
PA	Propionic (Propanoic) acid
BA	Butyric (Butanoic) acid
CA	Caproic (Hexanoic) acid
LLOQ	Lower limit of quantification
QC	Quality control sample(s)

## References

[1] Xiong R.G., Zhou D.D., Wu S.X., Huang S.Y., Saimaiti A., Yang Z.J., Shang A., Zhao C.N., Gan R.Y., Li H.B. (2022). Health Benefits and Side Effects of Short-Chain Fatty Acids. Foods.

[2] Zhang H., Yang H., Du S., Ren J., Qiao G., Ren J. (2025). Rutin ameliorates calcium oxalate crystal-induced kidney injury through anti-oxidative stress and modulation of intestinal flora. Urolithiasis.

[3] Barcutean L., Farczadi L., Manescu I.B., Imre S., Maier S., Balasa R. (2024). Short and Medium Chain Fatty Acids in a Cohort of Naïve Multiple Sclerosis Patients: Pre- and Post-Interferon Beta Treatment Assessment. Biologics.

[4] Pluznick J.L. (2016). Gut microbiota in renal physiology: Focus on short-chain fatty acids and their receptors. Kidney Int..

[5] Wu S., Bu X., Chen D., Wu X., Wu H., Caiyin Q., Qiao J. (2025). Molecules-mediated bidirectional interactions between microbes and human cells. NPJ Biofilm. Microbiomes.

[6] Meesters R.J., van Eijk H.M., ten Have G.A., de Graaf A.A., Venema K., van Rossum B.E., Deutz N.E. (2007). Application of liquid chromatography-mass spectrometry to measure the concentrations and study the synthesis of short chain fatty acids following stable isotope infusions. J. Chromatogr. B Anal. Technol. Biomed. Life Sci..

[7] Zeng M., Cao H. (2018). Fast quantification of short chain fatty acids and ketone bodies by liquid chromatography-tandem mass spectrometry after facile derivatization coupled with liquid-liquid extraction. J. Chromatogr. B Anal. Technol. Biomed. Life Sci..

[8] Chen Z., Gao Z., Wu Y., Shrestha R., Imai H., Uemura N., Hirano K.I., Chiba H., Hui S.P. (2019). Development of a simultaneous quantitation for short-, medium-, long-, and very long-chain fatty acids in human plasma by 2-nitrophenylhydrazine-derivatization and liquid chromatography-tandem mass spectrometry. J. Chromatogr. B Anal. Technol. Biomed. Life Sci..

[9] Song H.E., Lee H.Y., Kim S.J., Back S.H., Yoo H.J. (2019). A Facile Profiling Method of Short Chain Fatty Acids Using Liquid Chromatography-Mass Spectrometry. Metabolites.

[10] Darlington D.N. (2025). Conjugation of Short-Chain Fatty Acids to Bicyclic-Amines for Analysis by Liquid Chromatography Tandem Mass Spectroscopy. Molecules.

[11] Lee D., Kerry M.S. (2024). A simple liquid chromatography method running in dual modes for quantification of short and medium chain fatty acids. J. Chromatogr. A.

[12] Ng D.Z.W., Lee S.X.Y., Ooi D.S.Q., Ta L.D.H., Yap G.C., Tay C.J.X., Huang C.H., Tham E.H., Loo E.X.L., Shek L.P.C. (2023). Sensitive LC-MS/MS method for the temporal profiling of bile acids, fatty acids and branched-chain alpha-keto acids in maternal plasma during pregnancy and cord blood plasma at delivery. Clin. Chim. Acta.

[13] Wang X., Ruan H., Zong Z., Mao F., Wang Y., Jiao Y., Xu L., Yang T., Li W., Liu X. (2021). A simulated strategy for analysis of Short- to Long- chain fatty acids in mouse serum beyond chemical standards. J. Chromatogr. B Anal. Technol. Biomed. Life Sci..

[14] Aydoğan C. (2020). Recent advances and applications in LC-HRMS for food and plant natural products: A critical review. Anal. Bioanal. Chem..

[15] (2018). Bioanalytical Method Validation—Guidance for Industry.

[16] (2011). Guideline on Bioanalytical Method Validation.

[17] Nagatomo R., Ichikawa A., Kaneko H., Inoue K. (2024). Comparison of 3-nitrophenylhydrazine, O-benzyl hydroxylamine, and 2-picolylamine derivatizations for analysis of short-chain fatty acids through liquid chromatography coupled with tandem mass spectrometry. Anal. Sci..

[18] Chalova P., Tazky A., Skultety L., Minichova L., Chovanec M., Ciernikova S., Mikus P., Piestansky J. (2023). Determination of short-chain fatty acids as putative biomarkers of cancer diseases by modern analytical strategies and tools: A review. Front. Oncol..

[19] Li C., Liu Z., Bath C., Marett L., Pryce J., Rochfort S. (2022). Optimised Method for Short-Chain Fatty Acid Profiling of Bovine Milk and Serum. Molecules.

[20] Chen Z., Wu Y., Shrestha R., Gao Z., Zhao Y., Miura Y., Tamakoshi A., Chiba H., Hui S.-P. (2019). Determination of total, free and esterified short-chain fatty acid in human serum by liquid chromatography-mass spectrometry. Ann. Clin. Biochem..

[21] Cas M.D., Paroni R., Saccardo A., Casagni E., Arnoldi S., Gambaro V., Saresella M., Mario C., La Rosa F., Marventano I. (2020). A straightforward LC-MS/MS analysis to study serum profile of short and medium chain fatty acids. J. Chromatogr. B-Anal. Technol. Biomed. LIFE Sci..

[22] Jaochico A., Sangaraju D., Shahidi-Latham S.K. (2019). A rapid derivatization based LC-MS/MS method for quantitation of short-chain fatty acids in human plasma and urine. Bioanalysis.

[23] Vagaggini C., Brai A., Bonente D., Lombardi J., Poggialini F., Pasqualini C., Barone V., Nicoletti C., Bertelli E., Dreassi E. (2023). Development and validation of derivatization-based LC-MS/MS method for quantification of short-chain fatty acids in human, rat, and mouse plasma. J. Pharm. Biomed. Anal..

[24] Mayo-Martínez L., Lorenzo M.P., Martos-Moreno G.Á., Graell M., Barbas C., Rupérez F.J., Argente J., García A. (2024). Short-chain fatty acids in plasma and feces: An optimized and validated LC-QqQ-MS method applied to study anorexia nervosa. Microchem. J..

[25] Saha S., Day-Walsh P., Shehata E., Kroon P.A. (2021). Development and Validation of a LC-MS/MS Technique for the Analysis of Short Chain Fatty Acids in Tissues and Biological Fluids without Derivatisation Using Isotope Labelled Internal Standards. Molecules.

[26] Shafaei A., Vamathevan V., Pandohee J., Lawler N.G., Broadhurst D., Boyce M.C. (2021). Sensitive and quantitative determination of short-chain fatty acids in human serum using liquid chromatography mass spectrometry. Anal. Bioanal. Chem..

[27] Song W.-S., Park H.-G., Kim S.-M., Jo S.-H., Kim B.-G., Theberge A.B., Kim Y.-G. (2020). Chemical derivatization-based LC-MS/MS method for quantitation of gut microbial short-chain fatty acids. J. Ind. Eng. Chem..

[28] Li S., Xiao Q., Sun J., Li Z., Zhang M., Tian Y., Zhang Z., Dong H., Jiao Y., Xu F. (2024). A new chemical derivatization reagent sulfonyl piperazinyl for the quantification of fatty acids using LC-MS/MS. Talanta.

[29] Barcutean L., Maier S., Burai-Patrascu M., Farczadi L., Balasa R. (2024). The Immunomodulatory Potential of Short-Chain Fatty Acids in Multiple Sclerosis. Int. J. Mol. Sci..

